# Psychometric Evaluation of the BFI-10 and the NEO-FFI-3 in Indian Adolescents

**DOI:** 10.3389/fpsyg.2019.01057

**Published:** 2019-05-09

**Authors:** Roshin Kunnel John, Boby Xavier, Anja Waldmeier, Andrea Meyer, Jens Gaab

**Affiliations:** ^1^Division of Clinical Psychology and Psychotherapy, Faculty of Psychology, University of Basel, Basel, Switzerland; ^2^Division of Clinical Psychology and Epidemiology, Faculty of Psychology, University of Basel, Basel, Switzerland

**Keywords:** five-factor model, BFI-10, NEO-FFI-3, psychometric evaluation, Indian adolescents

## Abstract

The Five-Factor Model (FFM) is one of the most commonly examined constructs of personality across cultures in recent times. However, there is a lacuna of evidence for the suitability of FFM measures for Indian adolescent school students below the age of 17 years. We carried out two independent studies for the psychometric evaluation of the measures BFI-10 and NEO-FFI-3 on Indian adolescent school students. Both studies examined two socio-culturally distinct linguistic groups of secondary and senior secondary school students with a total sample of *N* = 1117 students. There was very limited support for a five-factor solution in both cases. Model fit was poor when applying FFM measures to our samples, whether applying confirmatory factor analysis or exploratory structural equation models. The results provide evidence against using adult personality measures with adolescents without separate psychometric validation and applying the Western age norms to Indian students without considering that the process of personality consolidation during adolescence may not be identical across cultures.

## Introduction

The scientific examination of personality is driven by the aim of identifying and predicting patterns of individuals’ thinking, feeling, and behaving based on the assumption that these patterns are relatively stable and universal amid socio-cultural diversities. Extensive cross-cultural research in the last three decades has come to establish the Five-Factor Model (FFM) of personality, often referred to as the Big Five with its domains *Neuroticism, Extraversion, Openness to experience, Agreeableness*, and *Conscientiousness* (McCrae and John, 1992; Goldberg, 1993; McCrae and Costa, 1997; John et al., 2008). Research on the FFM has led to the development and modifications of two broad sets of measures, i.e., the *Big Five Inventories* (BFI-44, BFI-10, and BFI-2) and the *NEO Inventories* (NEO-PI, NEO-PI-R, NEO-FFI, NEO-PI-3, and NEO-FFI-3). The two most commonly used NEO inventories are the Revised NEO Personality Inventory (NEO-PI-R) and the NEO-Five- Factor Inventory (NEO-FFI) developed by Costa and McCrae (1992). The NEO-FFI-3 (McCrae and Costa, 2007), which is a revision of the NEO-FFI aimed at better readability especially for respondents who are not native English speakers. Across studies, good Cronbach’s alpha reliability has been demonstrated for its subscales (McCrae and Costa, 2007; Marjanovic et al., 2015). Furthermore, and independent of the NEO Inventories, the Big Five Inventory-44 (BFI-44) was developed as a time-efficient measure of the FFM (John et al., 1991; Soto and John, 2009). The BFI-44 has been translated into 28 languages and its structure has been replicated in 56 nations (Schmitt et al., 2007). The pursuit of brevity resulted in the BFI-10 (Rammstedt and John, 2007), which consists of just two items to represent each of the five core personality dimensions. Brief as it is, the BFI-10 offers an efficient assessment and has been validated in the United States and Germany in the respective languages (Rammstedt and John, 2007; Rammstedt et al., 2013). The Next Big Five Inventory (BFI-2) is a new 60 item measure of the five-factors (Soto and John, 2017).

The diverse FFM measures have gathered a strong evidence base in the Western societies where they originated (Goldberg, 1993; Costa and McCrae, 1997; McCrae and Costa, 2010) and their psychometric qualities were reproduced in a number of cross-cultural studies incorporating different societies on most continents (McCrae and Terracciano, 2005; Schmitt et al., 2007; see also Allik and Realo, 2017). However, a few studies could not replicate the five-factor structure in non-Western societies (e.g., Cheung et al., 2011; Gurven et al., 2013; Zecca et al., 2013). Gurven et al. (2013) argued that, even in cross-cultural studies the participation was often limited to the educated urban population and that the FFM is yet to have evidence for indigenous and often illiterate societies (Gurven et al., 2013). Besides, these cross-cultural studies have observed small variances from the United States normative data in the developing countries (McCrae and Terracciano, 2005; Schmitt et al., 2007).

Five-factor model studies in Europe and the United States have demonstrated the five-factor structure in adolescents with the lowest age cut-off of 12 years in the normative data for the Big Five as well as the NEO inventories (Costa et al., 2000; McCrae et al., 2002; McCrae and Costa, 2004; O’Connor and Paunonen, 2007; Klimstra et al., 2009). However, it has been observed that adolescents below 16 years of age are likely to provide a relatively lower quality of the self-reported personality trait structure (Allik and Realo, 2017).

With respect to the FFM research in India, so far, around 35 published studies have employed either the NEO inventories or the Big Five inventories in the Indian context. However, most of these studies were conducted on adults and college students, and none of these studies have reported the psychometric values of the FFM measures on adolescent school students. The NEO Inventories were used in 21 out of the 35 FFM studies. Of these, three studies addressed psychometric evaluation of the NEO inventories. Lodhi et al. (2002) and Singh (2009) examined NEO-PI-R, and Piedmont and Braganza (2015) studied NEO-PI-3. Whereas Singh (2009) studied young adults in the age range of 18–25 years, the other two studies were conducted on adult samples in the age range of 18–60 years. All the three studies found acceptable alpha reliability values ranging from 0.73 to 0.93 for the five-factors, and the factor structures were replicated. However, it was found that the scores for factors Extraversion and Agreeableness aligned differently from the pattern in the United States normative data. Four FFM studies in which the NEO-FFI was used with adults, reported acceptable alpha reliability values ranging from 0.63 to 0.88 (Dubey et al., 2010; Madnawat and Mehta, 2012; Dabke, 2014; Magan et al., 2014). However, in a study on postgraduate students, Joshi and Thingujam (2009) reported inadequate alpha reliability values for the NEO-FFI. The obtained psychometric values of the FFM measures are not mentioned in the other studies including 12 studies which administered the NEO-FFI/NEO-PI-R on college students or working youth of 18–30 years of age (Chaturvedula and Joseph, 2007; Pavitra et al., 2007; Sharma et al., 2010; Fazeli, 2012; Sushma et al., 2015; Sharma and Gill, 2016; Srivastava and Mishra, 2016; Gupta, 2017; Mandal, 2017; Rita, 2017; Ullah, 2017; Abbas and Khan, 2018). Singh and Ullah (2016) used NEO-PI-R with adolescent school students, but did not mention the obtained alpha reliability values.

Unlike the NEO inventories, there are no published Indian studies on the psychometric evaluation of any version of the BFI. The Big Five Inventories were used in 14 out of the 35 FFM studies. In eight of them, the BFI-44 were administered with working youth or college students (Andi, 2012; Subramanian et al., 2012; Aggarwal et al., 2014; Joshi and Bhardwaj, 2016; Patki and Abhyankar, 2016; Saini et al., 2016; Thurackal et al., 2016; Parekh, 2018), and in two other studies of young adults the BFI-10 was used (Varghese and Raj, 2014; Mahajan et al., 2017). There were also two studies in which the BFI-44 was used with school students (Kumari and Sharma, 2016; Salve et al., 2017). Unfortunately, none of these 12 studies mentioned the obtained psychometric values of the BFI-44 or BFI-10. In the international cross-cultural study of Schmitt et al. (2007) 100 Indian college students were included. However, separate scores for the Indian subgroup are not mentioned in the study. Finally, Singh and Yu (2010) who reported the obtained alpha reliability values of the BFI-44, which was administered on college students aged 18–27 years, found that none of the five-factors had acceptable alpha reliability.

Thus, no study has yet reported the psychometric values of NEO-FFI-3 and BFI-10 in India and no study has yet examined the applicability of the FFM for the Indian adolescents. Unfortunately, the few published studies (e.g., Kumari and Sharma, 2016; Singh and Ullah, 2016) in which FFM measures were administered on Indian adolescent school students, do not report the psychometric values. Though the applicability of FFM measures for Indian adults have been demonstrated in some studies, a few studies raise questions. Besides, establishing the applicability of the measures on adults does not automatically make them reliable and valid for the adolescents. We therefore set out to evaluate the psychometric properties of BFI-10 and NEO-FFI-3 on Indian adolescents in two independent and multisite studies. We aimed at replicating the five-factor structure on school-going Indian adolescents in the age group of 15–18 years, and to examine the utility of time-effective FFM measures for this age-group in the Indian context.

India is unique in its complex ethnic and cultural diversity. The seven-decade old nation retains a diversity of over 10,000 distinct ethnic communities (Fearon, 2003; Roy, 2011), and 1,369 languages (Census of India, 2011). The Indian “societies” have been described as predominantly collectivistic and interdependent (Sinha et al., 2001) and hence socio-culturally distinct from the individualistic Anglo-American societies where the FFM was developed. We set out to examine two socio-culturally distinct and geographically distant linguistic groups of adolescents from two Indian states: Madhya Pradesh from North India and Kerala from South India. Kerala ranks as one of the best among Indian states on social developmental and quality of life indicators, whereas Madhya Pradesh stands slightly below the average rates for India (Census of India, 2011). Kerala has the highest literacy rate (94%) among Indian states, much higher than Madhya Pradesh (70%) and the national literacy rate (74%). Kerala boasts of equal educational opportunity for male and female children as compared to the other Indian states including Madhya Pradesh where females lag behind. Kerala also has the highest life-expectancy and infant mortality rates, while Madhya Pradesh is close to India’s average rates (Census of India, 2011). Keralites speak Malayalam as their mother tongue whereas the mother tongue of Madhya Pradesh is Hindi which is India’s most common language. We used native translations of the measures, and sought to incorporate cultural diversity by including self-report of religion and caste affiliation as well as school records of the governmental class stratification of the students.

## Materials and Methods

We explored the psychometric properties of the BFI-10 and the NEO-FFI-3 personality inventories in two separate studies with two independent groups of adolescent school students. We used single-group cross-sectional designs for both the studies.

### Participants

BFI-10 study was conducted on a sample of 679 students and the NEO-FFI-3 study on 438 students. For both studies, participants were in the age range of 15–18 years. Both studies had linguistic subgroups with reference to the two States where the study was done, i.e., Kerala in South India and Madhya Pradesh in North India. Details of the demographic information on the two studies are given in Table 1.

**Table 1:** Participants demographic details.

Age (range and M in years)		BFI-10 15–18, *M* = 16.5, SD = 0.7		NEO-FFI-3 15–18, *M* = 15.9, SD = 0.7	
		
Gender (N/%)		Women	Men	Women	Men
		379/55.8	300/44.2	202/46.1	236/53.9
Standard- levels		N	%	N	%
BFI-10 (X/XI/XII)		356/257/66	52.4/37.8/9.7	143/295	32.6/67.4
NEO-FFI-3 (XI/XII)					
Linguistic subgroups (N/%)	South India (Kerala)	404/59.5		187/42.7	
	North India (Madhya Pradesh)	275/40.5		251/57.3	
Governmental Class (N/%)	SC/ST	29/4.3		34/7.8	
	OBC	322/47.4		187/42.7	
	General	328/48.3		217/49.5	


*NB: SC/ST, scheduled cast/scheduled tribes; OBC, other backward class.*

### Measures

For the BFI-10 study, we used the 10-item short-version of the Big Five Inventory (BFI-10, Rammstedt and John, 2007). The BFI-10 has five subscales with two bidirectional items for each of the big-five personality factors. The items are rated on a five-point Likert scale wherein the subjects choose from responses ranging from “strongly disagree to strongly agree.” For the NEO-FFI-3 study, we used the NEO-FFI-3 form S – Adolescent, Self-Report which consists of 60 items, with 12 items each for the big-five personality factors (McCrae and Costa, 2010). The NEO-FFI-3 is a revision of the NEO-FFI (Costa and McCrae, 1992) in which 15 of the 60 items have been revised to improve readability and psychometric properties. The measure uses a five-point Likert scale of responses ranging from “strongly disagree to strongly agree.”

### Procedure

For both the BFI-10 and the NEO-FFI-3 measures, translated versions of Hindi and Malayalam were used. Questionnaires were first translated into the regional Indian languages, i.e., Hindi for students in Madhya Pradesh and Malayalam for students in Kerala, by two Indian native school teachers and translated back into English by two different Indian native school teachers. These final English versions were then checked by the principal investigator (JG) in Switzerland through comparison of the back-translations with original English versions of the BFI-10 and NEO-FFI-3.

The research project was submitted to the Cantonal Ethics Committee (Basel-Stadt and Basel-Land), which positively acknowledged the study protocol and informed consent forms but stated that the approval needed to be assessed by local authorities. We therefore also obtained the necessary permission from the respective school management trusties as well as the permission of school principals. Prior to data collection, written informed consent was obtained from participants in the age range of 17–18 years as well as parents of participants in the age range of 15–16 years. Also, the assent was obtained from participants in this age group of 15–16 years.

The BFI-10 study was conducted between April and June 2014 and the NEO-FFI-3 study between February and March 2016. Secondary and senior secondary students from six schools were recruited from two states of India, i.e., Hindi speaking students from Madhya Pradesh in North India and Malayalam speaking students from Kerala in South India. Participants for both the studies were recruited from the same schools.

Questionnaires and the respective instructions for use were sent to the instructed contact persons in India who supervised the study procedure. School teachers were assigned to collect data from participants allotted to their supervision. For all participants, the demographic variables such as age, gender, religion, class of study, and caste affiliation were elicited by self-report. The Governmental class stratification, which comprises General Class, Scheduled Castes, and Scheduled Tribes, and Other Backward Class was obtained from school records. Students were asked to complete the questionnaires according to the written instructions. Participants of BFI-10 study were given 15 min and participants of NEO-FFI-3 study were given 40 min to complete the questionnaires. The completed questionnaires were collected and sent back to Switzerland for scoring and analysis.

In both studies, a double-check process was carried out upon data entry. Subsequently, data validation was carried out according to the administration and scoring instructions in the manuals. In BFI-10, the data of 6 students were deleted because of incomplete demographic information. For NEO-FFI-3, in the case of ten or more missing items, the data was considered invalid as per the instructions in the manual (McCrae and Costa, 2010). In consequence, the data of 12 students were deleted, of which 10 students had ten or more missing items and two students did not provide demographic information. The full information maximum likelihood (FIML) algorithm was used for managing missing data in all analyses (Baraldi and Enders, 2010). The overall number of participants in the two studies were *N* = 1135 (BFI-10: *N* = 685 and NEO-FFI-3: *N* = 450). After the removal of 18 incomplete data from both the studies (BFI-10: *n* = 6 and NEO-FFI-3: *n* = 12), the overall sample for analysis was 1117.

#### Statistical Analyses

Statistical analyses were performed using Mplus, version 8 (Muthén and Muthén, 2017). For each of the two samples, confirmatory factor analyses (CFA) as well as exploratory structural equation models (ESEM) were set up to test the suitability of the two FFM measures, BFI-10, and NEO-FFI-3. As estimator we used maximum likelihood with robust standard errors and for ESEM the rotation method was the oblique goemin (Costello and Osborne, 2005). We also calculated internal consistency for each of the five factors using Cronbach’s alpha.

Using ESEM, we further explored whether factor solutions lower or higher than five might better explain our data. Because of their purely exploratory nature, these additional analyses are only briefly reported in text.

## Results

### BFI-10 Study

For the BFI-10 study, the assumed five-factor solution did not converge for either CFA or ESEM. For CFA, fixing factor variances to one while freeing first indicator loadings of each factor did not fix the problem. Fixing the loading of one item (item 4) to a predefined value lead to model convergence but the latent variable covariance matrix was not positive with several negative estimates of residual variances (item 4, item 6, and item 8). Similarly, for ESEM estimates of several residual variances (item 2, item 3) were negative and standard errors of parameter estimates could not be computed. Therefore, no fit indices are presented.

We further explored whether lower or higher factor solution of ESEM lead to a better model fit. Unlike the four-factor solution, which also involved model fit problems (i.e., negative residual variances), a three-factor solution returned a more or less reasonable model fit. Fit indices for this model were (see Table 2): χ^2^(18) = 144.2, *p* = <0.001, RMSEA = 0.10 *p*_(RMSEA)_ < 0.001, CFI = 0.918, TLI = 0.796, SRMR = 0.035. Higher factor solutions suffered from the same model convergence problems as the five-factor solution.^1^

**Table 2:** Standardized parameter estimates for the ESEM *3 factor* solution of the BFI-3.

Item	Scale	1	2	3
1	E	0.075	0.109	0.102
6	E	0.191	0.726	0.020
2	A	0.904	0.426	0.013
7	A	1.047	0.007	-0.179
3	C	0.247	-0.101	-0.517
8	C	0.264	0.461	-0.228
4	N	0.004	-0.494	0.581
9	N	0.030	-0.018	0.753
5	O	0.003	1.093	0.013
10	O	-0.004	0.976	-0.031


The reliability analysis for the five subscales of BFI-10 in the overall sample yielded mixed results (see Table 3), with unacceptably low alpha reliabilities for Neuroticism, Extraversion, and Conscientiousness and acceptable alpha reliabilities for Openness and Agreeableness. Openness and Agreeableness showed a strong positive correlation (Spearman’s rho = 0.58, *p* = 0.001). These overall results appeared not to be influenced by age as a similar picture was observed in separate analyses with younger (15–16) and older (17–18) subgroups of students. The detailed description of the reliability analysis of BFI-10 is shown in Table 3.

**Table 3:** Internal consistency of overall sample and linguistic subgroups.

		Cronbach’s alpha	Age 15–16/17–18 years	KL (*n* = 404 + 187)	MP (*n* = 275 + 251)
BFI 10	Neuroticism	0.45	0.36/0.53	0.57	0.43
	Extraversion	0.44	0.37/0.48	0.43	0.30
	Openness	0.76	0.75/0.73	0.55	0.40
	Agreeableness	0.78	0.78/0.77	0.57	0.63
	Conscientiousness	0.43	0.34/0.53	0.53	0.38
NEO-FFI-3	Neuroticism	0.52	0.53/0.62	0.42	0.59
	Extraversion	0.14	0.15/0.17	0.16	0.18
	Openness	0.40	0.35/0.57	0.52	0.25
	Agreeableness	0.50	0.46/0.64	0.55	0.47
	Conscientiousness	0.71	0.73/0.72	0.74	0.70


*NB: KL, Kerala; MP, Madhya Pradesh.*

Internal consistencies are also presented by age groups (15–16 versus 17–18 years) and by linguistic subgroups (Malayalam speaking, from Kerala, KL versus Hindi speaking, from Madhya Pradesh, MP), again showing poor values (Table 3).

### NEO-FFI-3 Study

For the NEO-FFI-3 study, as for the BFI-10 study, the assumed five-factor solution of the CFA did not converge. Fixing factor variances to one while freeing first indicator loadings of each factor lead to model convergence, but the model fit was poor [χ^2^(1700) = 3261.8, *p* = <0.001, RMSEA = 0.046 *p*_(RMSEA)_ = 0.998, CFI = 0.454, TLI = 0.431, SRMR = 0.071] (see Table 4). Though the five-factor solution of the ESEM converged, the model fit was also poor [χ^2^(1480) = 2289.5, *p* = <0.001, RMSEA = 0.035 *p*_(RMSEA)_ = 1.00, CFI = 0.717, TLI = 0.661, SRMR = 0.043] (see Table 4).

**Table 4:** Standardized parameter estimates for the CFA and ESEM solution of the NEO-FFI-3.

		CFA					ESEM				
			
Item	Scale	1	2	3	4	5	1	2	3	4	5
1	N	0.037					0.055	**-0.464**	-0.008	-0.315	0.069
6	N	**0.359**					**0.356**	0.219	-0.104	0.188	0.182
11	N	**0.471**					**0.504**	0.110	0.085	0.086	0.076
16	N	0.194					0.172	**-0.351**	-0.119	0.011	0.171
21	N	**0.636**					**0.645**	0.005	0.147	**0.404**	0.060
26	N	**0.482**					**0.520**	0.066	0.004	0.115	-0.077
31	N	0.159					0.115	-0.260	-0.097	-0.040	-0.062
36	N	**0.584**					**0.560**	0.049	-0.223	0.014	-0.009
41	N	**0.593**					**0.487**	-0.075	-0.237	0.184	-0.036
46	N	**-0.347**					**-0.358**	-0.084	-0.131	0.072	0.049
51	N	**0.525**					**0.569**	0.011	0.209	0.022	**-0.389**
56	N	**0.621**					**0.614**	-0.012	-0.016	0.017	-0.144
2	E		**0.359**				0.108	0.292	0.080	-0.119	0.012
7	E		0.251				0.000	0.382	-0.090	-0.067	-0.110
12	E		0.180				-0.118	0.111	-0.038	-0.153	-0.162
17	E		**-0.540**				-0.076	-0.495	-0.079	-0.080	-0.007
22	E		0.120				-0.020	0.081	-0.125	**0.355**	0.273
27	E		**0.444**				**-0.445**	0.302	0.041	0.080	0.127
32	E		**0.304**				0.010	0.238	-0.078	-0.249	0.247
37	E		0.277				-0.049	0.332	0.019	**-0.447**	-0.046
42	E		**0.446**				-0.195	**0.463**	-0.010	0.150	-0.022
47	E		0.074				0.024	0.164	-0.126	0.025	0.146
52	E		0.087				0.021	-0.119	0.256	-0.193	0.165
57	E		-0.151				-0.164	-0.121	-0.125	-0.157	-0.204
3	O			0.184			0.293	-0.042	0.216	**-0.442**	0.136
8	O			0.204			0.138	0.154	0.129	0.011	0.138
13	O			**0.393**			0.069	0.213	0.164	0.033	0.222
18	O			-0.183			-0.107	0.017	-0.033	0.001	-0.223
23	O			**0.514**			-0.094	-0.098	**0.431**	0.008	0.028
28	O			**0.322**			**-0.415**	-0.043	0.087	0.013	**0.387**
33	O			0.222			-0.270	-0.142	0.028	0.114	0.222
38	O			0.027			0.163	0.202	-0.110	-0.209	0.167
43	O			**0.642**			-0.025	-0.023	**0.488**	-0.007	0.073
48	O			**0.333**			-0.062	0.146	0.214	-0.023	0.019
53	O			**0.323**			0.019	-0.062	0.209	-0.012	**0.312**
58	O			0.195			0.064	-0.096	0.197	0.006	0.055
4	A				**0.372**		0.057	0.108	**0.375**	0.008	-0.041
9	A				**0.524**		-0.160	-0.024	**0.365**	**0.478**	-0.090
14	A				0.262		**-0.508**	0.275	**0.078**	0.091	-0.207
19	A				**0.720**		-0.022	**-0.404**	**0.735**	0.070	-0.167
24	A				-0.021		0.014	-0.102	0.007	0.218	**-0.341**
29	A				**0.549**		0.171	-0.069	**0.547**	0.165	-0.148
34	A				**0.337**		-0.048	0.191	**0.332**	-0.025	-0.094
39	A				0.133		**-0.384**	0.227	-0.023	0.105	**-0.312**
44	A				**0.323**		-0.045	0.023	0.262	0.234	0.044
49	A				0.026		0.212	0.283	-0.016	**0.362**	0.219
54	A				0.138		0.030	-0.014	0.134	**0.369**	-0.124
59	A				**0.505**		-0.195	-0.228	**0.381**	0.093	-0.045
5	C					**0.392**	0.032	0.081	**0.424**	-0.090	-0.047
10	C					**0.494**	-0.153	0.145	**0.422**	-0.010	-0.033
15	C					**0.301**	-0.299	-0.024	0.213	**-0.637**	-0.037
20	C					**0.604**	0.011	0.158	**0.532**	-0.053	0.068
25	C					**0.580**	-0.081	0.114	**0.431**	-0.015	0.213
30	C					**0.516**	-0.295	-0.111	**0.378**	0.180	0.259
35	C					**0.507**	0.064	0.102	**0.463**	-0.213	0.053
40	C					**0.435**	-0.048	0.288	**0.298**	-0.076	0.110
45	C					**0.255**	-0.289	0.143	0.010	0.282	0.278
50	C					**0.534**	-0.012	-0.024	**0.442**	-0.068	**0.323**
55	C					**0.486**	**-0.441**	0.077	0.221	0.047	0.252
60	C					**0.355**	-0.042	0.056	0.238	0.063	0.283


*Items above 0.30 are bold (Brown, 2006).*

Exploring lower factor solutions for ESEM lead to worse model fit, while higher factor solutions (6- and 7-factors) hardly improved model fit compared to the five-factor solution.

Internal consistencies for the personality domains in the total sample as well as linguistic subgroups were mostly unacceptable or poor, with the exception of Conscientiousness (Table 3). For age subgroups, (15–16 versus 17–18 years) internal consistency was better for older students especially on Neuroticism and Agreeableness (Table 3).

## Discussion

We conducted two independent studies exploring the factor structure and reliability of BFI-10 and NEO-FFI-3 with two socio-culturally distinct linguistic groups of Indian adolescent school students. There was very limited support for a five-factor solution for both measures in our samples. Model fit was poor when applying CFA or ESEM for both BFI-10 (Rammstedt and John, 2007) and NEO-FFI-3 (McCrae and Costa, 2007). Acceptable internal consistency was found only for the subscales, Openness to experience and Agreeableness in the BFI-10 and Conscientiousness in the NEO-FFI-3.

On BFI-10, while five-factor solution did not converge on CFA and ESEM, a three-factor solution of ESEM lead to a better model fit. However, only the items for Neuroticism clustered, and a meaningful pattern could not be identified on the other two factors (Table 2). This seems to suggest that the ultra-brief BFI-10 measure may not be applicable in its current form for Indian adolescent school students.

On NEO-FFI-3, while a five-factor solution converged on CFA and ESEM with a poor model fit, lower or higher factor solutions did not lead to a better model fit. All the 12 items for Consciousness clustered on CFA. For Neuroticism, three of the 12 items had poor factor loading and these were items with reverse scoring (Table 4). Other subscales did not emerge clearly. This points to the need for modification of the measure in order to examine the applicability of FFM for this target population.

Whereas previous studies have not examined the factor structure and reliability of the FFM measures in Indian adolescents, in two Indian studies of college students (18–27 years of age) which used FFM measures, the alpha reliability values obtained were similar to the results in our studies. On BFI-44, Singh and Yu (2010) found that none of the five-factors had acceptable internal consistency (Cronbach’s alpha coefficients: Conscientiousness α = 0.52, Neuroticism α = 0.54, Openness α = 0.54; Agreeableness α = 0.64 and Extraversion α = 0.67). These values show some resemblance to the results in our BFI-10 study. Similarly, Joshi and Thingujam (2009) reported unacceptable internal consistency for the NEO-FFI subscales (Neuroticism α = 0.61, Openness α = 0.49; Agreeableness α = 0.51 and Extraversion α = 0.63), except Conscientiousness (α = 0.71). These values closely resemble the obtained alpha reliabilities in our NEO-FFI-3 study.

Our results, combined with the fact that previous studies have not established the suitability of FFM measures for Indian school students, points to three possibilities regarding the applicability of these measures in the Indian context. First, India’s cultural difference from the Western societies may have contributed to the results. Even if the five-factor theory is universally applicable, the FFM measures may not be applicable as such in all non-Western cultures and may need modification to fit the Indian socio-cultural context. Second, the Indian society is culturally so diverse that the measures tested with one segment of the society may not be automatically applicable and relevant as such for another segment which is different in socio-cultural and geographical-linguistic aspects. Third, the available evidence of applicability of some of the FFM measures for Indian adults may not indicate applicability for Indian adolescents.

With respect to the first possibility, there is empirical evidence to suggest that the FFM measures which have their roots in the individualistic Western society, are less suited to describe personality in some of the collectivistic societies of Africa and Asia (Ashton and Lee, 2007; Vogt and Laher, 2009; Allik et al., 2013; Gurven et al., 2013; Laher, 2013; Valchev et al., 2013; Allik and Realo, 2017; Singh and De Raad, 2017). For example, Laher (2008) based on his review of NEO-PI-R studies in Africa observed that evidence for the structural equivalence of NEO-PI-R across cultures was lacking with respect to the African context. Similarly, Gurven et al. (2013) could not replicate the BFI-44 in an indigenous Bolivian sample. In some FFM studies in non-Western cultures, the factors Extraversion and Agreeableness were not clearly differentiated (e.g., Rolland, 2002; Ortiz et al., 2007). Moreover, personality traits captured through descriptive adjectives may not exactly relate to the same construct across cultures (Vogt and Laher, 2009). For instance, in our study, the Malayalam equivalent for the adjective “worrier” on the item 1 of NEO-FFI-3 (“I am not a worrier”), had alternative shades of meaning as “a problematic person.” Similarly, all aspects of personality in collectivistic cultures may not be represented in the five-factors. Zhou et al. (2009), for instance, have given evidence of a seven-factor personality structure in Chinese populations. Other Asian studies have provided evidence for additional domains like “interpersonal relatedness” which are not adequately captured in the FFM (Cheung, 2004; Ashton and Lee, 2007; Cheung et al., 2008). In India, Singh et al. (2013) gave evidence for a three-factor personality structure linked to the ancient upanishadic “trigunas” and suggested that the FFM did not adequately describe the Hindi speaking participants’ personality (see also Singh, 2016; Singh and De Raad, 2017). Hence, there are reasons for not expecting that a particular number of trait dimensions would emerge in a non-Western culture when personality factors of an inventory are developed based on lexical usages of the native language, i.e., in an “emic” measure (Gurven et al., 2013).

The second possibility has to do with India’s complex socio-cultural diversity. Current Indian society is characterized by the coexistence of collectivism and individualism and may need multiple and divergent paradigms to define it (Sinha and Tripathi, 1994; Sinha et al., 2001). Allik and McCrae (2004) have observed that the Black and the White South Africans present with different personality profiles though they reside in the same geophysical location. In our study, we tried to evaluate the potential of the FFM to describe two distinct linguistic groups, namely, Malayalam speaking and Hindi speaking students. On NEO-FFI-3, both the groups had acceptable alpha reliability value for the factor Conscientiousness only, and the lowest alpha reliability values were found on the factor Extraversion. Differences in the alpha reliability values were also observed across the two groups. Openness had comparatively higher alpha reliability values for the Malayalam speaking students in both BFI-10 and NEO-FFI-3 measures. It has been observed that the factor Openness show relatively weak alpha reliability value in collectivistic and less developed countries (Piedmont et al., 2002). Kerala is relatively more “westernized” as compared to other states in India, and its scale of human development is comparable to that of some of the developed countries (Anisha and Praseetha, 2016). Hence, the difference of alpha reliability on this domain might be an indicator of the cultural difference of the two linguistic groups. Group differences on personality domains were not examined in this study because the factor subscales did not emerge as reliable.

Thirdly, this study raises questions about the applicability of the five-factor measures for the Indian adolescents. Although some of the studies using NEO Inventories have demonstrated evidence for the FFM in Indian adult sample, validating the measures on adults may not automatically make them reliable and valid for adolescents. Adolescent personality development in the predominantly collectivistic and interdependent Indian societies is likely to follow a trajectory different from that of the individualistic and personal agency based European-American societies where the FFM was developed (Chadda and Deb, 2013; Schwartz et al., 2012). Though the Western validation studies provide an age range beginning at 12 years, our findings point to the possibility that the secondary and senior secondary students may present with either a different personality profile or a poorly consolidated personality, or simply that the questionnaires are not able to capture personality in this population. Collectivistic Indian families foster social cohesion, role conformity and interdependence in children rather than self-direction and personal choice (Chadda and Deb, 2013; Savita et al., 2014; Arusubila and Subasree, 2016). The development of identity and personality in adolescence follows the maturity principle, wherein freedom and perceived autonomy facilitates seeking out social contexts conducive for building up dispositional attributes like the five-factors (Caspi et al., 2005). Hence, in India, personality consolidation in terms of developmental years may not be identical to that of the individualistic Western societies. This argument is strengthened by our finding that, when the NEO-FFI-3 data from the small subsample of students in the age range of 17–18 years was separately analyzed, the internal consistency considerably improved (especially for Neuroticism, Agreeableness). This strengthens the possibility that the measure is less suitable for the younger Indian adolescents.

A few methodological factors may also have contributed to the results of our study. Imported measures are likely to suffer from lack of item relevance in the local culture which may affect translation (Ashton et al., 1998). Allik et al. (2013) argue that poor fit of FFM in less industrialized population could be attributed to aspects of the data quality such as negative item bias. According to them, people in less developed countries who live in relative poverty are likely to mark negatively worded items differently from the positively worded items. Besides, the problem of “double negative” may occur when responding negatively to an item with a reverse score. For instance, responding to the item “I am not a worrier” (NEO- FFI-3, item 1) might pose a problem. If someone asks “Are you not a worrier?”, answering “No” would mean the person is not a worrier. However, responding negatively to the item “I am not a worrier” would give the opposite sense that the person is actually a worrier. A recent research by Suárez-Alvarez et al. (2018) shows that the use of reverse and regular items in Likert scales is a questionable practice. According to these authors, comprehension of reversed items needs better linguistic skills and hence favors participants with higher verbal abilities. Also, these items are likely to reduce response variability and the quality of psychometric properties (Suárez-Alvarez et al., 2018). Gurven et al. (2013) found that items with a reverse scoring were problematic for his indigenous Bolivian farmers and that removal of these items reduced response biases and improved the factor structure. It was observed in our study that the negatively worded items with reverse scoring such as the one mentioned above had poor factor loadings and contributed to lower alpha reliability values. Hence, modification of these items is likely to strengthen reliability of the measure and produce a better factor structure reflecting the FFM.

The use of self-rating poses yet another problem in adolescent studies. In this study, as in all previous Indian studies of FFM measures, self-rating questionnaire was used without rating by others. Objective report of teacher/parent/peer would strengthen the validity of self-rating by adolescents. Baker et al. (2004) used multi-rating to examine the convergent and discriminant validity of the five-factor personality measure for adolescents. They found that when it comes to investigating adolescent personality, self-rating was a weaker method, as compared to teacher rating and peer rating. Allik and Realo (2017) have proposed that objective rating is needed along with self-rating for participants who are below 16 years of age.

The present study calls for notice as the first evaluation of the psychometric properties of the big-five traits specifically on Indian adolescent school students. Compared to previous FFM Indian studies of psychometric evaluation, we used a larger sample, translated the measures to native languages and incorporated socio-cultural diversity of participants that enhance generalizability. We found that the FFM measures (BFI-10 and NEO-FFI-3) were not suitable as such for the Indian adolescent school students. A valid alternative factor structure did not emerge from our CFA and ESEM or additional analyses. The problem of using reverse items for school students, the confusion that may arise from items with “double negative,” and cultural factors that affect translation may have contributed to poor model fit and reliability of the FFM measures in our samples. Besides, the application of Western age norms on Indian students could be problematic since the process of personality consolidation during adolescence may not be identical across cultures. We expect that revision of certain items, such as those involving reverse scoring can lead to clearer patterns when assessing the structure of the FFM in this target population. The results provide important evidence against the practice of using adult personality measures on adolescents without separate psychometric validation. Future studies should address the scope of modifying FFM measures in order to make them valid and sensitive specifically for Indian school students.

## Ethics Statement

The research project was submitted to the Cantonal Ethics Committee (Basel-Stadt and Basel-Land), which positively acknowledged the study protocol and informed consent forms but stated that the approval needed to be assessed by local authorities. We therefore also obtained the necessary permission from the respective school management trustees as well as the permission of school principals. Prior to data collection, written informed consent was obtained from participants in the age range of 17–18 years as well as parents of participants in the age range of 15–16 years. Also, the assent was obtained from participants in this age group of 15–16 years.

## Author Contributions

JG, BX, AM, and AW designed the study. AM, RK, BX, AW, and JG analyzed the data. JG, AW, and RK prepared the manuscript.

## Conflict of Interest Statement

The authors declare that the research was conducted in the absence of any commercial or financial relationships that could be construed as a potential conflict of interest.

## Footnotes

1 TLI, Tuck Lewis index; CFI, comparative fit index; RMSEA, root mean square error of approximation; SRMR, standardized root means square residual.
